# A standardized crisis management model for self-harming and suicidal individuals with three or more diagnostic criteria of borderline personality disorder: The Brief Admission Skåne randomized controlled trial protocol (BASRCT)

**DOI:** 10.1186/s12888-017-1371-6

**Published:** 2017-06-15

**Authors:** Sophie I. Liljedahl, Marjolein Helleman, Daiva Daukantaité, Åsa Westrin, Sofie Westling

**Affiliations:** 1 Department of Psychology, Lund University, Box 213, SE-221 00 Lund, Sweden; 2 Department of Clinical Sciences, Lund, Psychiatry, Clinical Psychiatric Research Center, Lund University, Lund, Region Skane Sweden; 3 Dimence Group, Centre for Mental Health Care, Burgemeester Roelenweg 9, 8021 EV Zwolle, the Netherlands

**Keywords:** Brief admission Skåne, Self-harm, Suicide, Borderline personality disorder, BASRCT

## Abstract

**Background:**

Brief Admission is a crisis and risk management strategy in which self-harming and suicidal individuals with three or more diagnostic criteria of borderline personality disorder self-admit to hospital at times of increasing risk when other efforts to stay safe are failing. Standardized in the current randomized controlled trial, the intensity of Brief Admission Skåne is implemented in durations of three days, with a maximum frequency of three times a month. Brief Admission is integrated into existing treatment plans in advance of crises to prevent reliance on general psychiatric admissions for risk management, as these may be lengthy, unstructured, and of uncertain therapeutic value.

**Methods/design:**

The overall objective of the Brief Admission Skåne randomized controlled trial is to determine if Brief Admission can replace general psychiatric admission for self-harming and suicidal individuals with complex mental illness at times of escalating risk. Other objectives of the study are to evaluate whether Brief Admission increases daily functioning and enhances coping, reduces psychiatric symptoms including frequency and severity of self-harm and suicidal behaviours. A final objective is to determine if Brief Admission is an effective crisis management model for this population. Participants are randomized at an individual level to either Brief Admission Skåne plus Treatment as Usual or Treatment As Usual. Based on a priori power analyses, *N* = 124 participants will be recruited to the study. Data collection is in progress, and will continue until June 2018. All participant data are single-blinded and will be handled with intention-to-treat analysis.

**Discussion:**

Based on the combined clinical experience of our international research group, the Brief Admission Skåne randomized controlled trial upon which the current protocol is based represents the first initiative to standardize, implement and evaluate Brief Admission amongst self-harming and suicidal individuals, including those with borderline traits. Objectively measuring protocol fidelity and developing English-language Brief Admission study protocols and training materials are implementation and dissemination targets developed in order to facilitate adherent international export of Brief Admission Skåne.

**Trial registration:**

NCT02985047. Registered November 25, 2016. Retrospectively registered.

**Electronic supplementary material:**

The online version of this article (doi:10.1186/s12888-017-1371-6) contains supplementary material, which is available to authorized users.

## Background

As stated by the National Institute for Health and Care Excellence [1] “The experience of care for people who self-harm is often unacceptable.” There is a misconception even amongst some health care providers that individuals who self-harm are choosing their suffering. This creates stigma regarding self-harm, about which individuals with lived experience are often well aware. Similar experiences with respect to stigma have been reported by people diagnosed with borderline personality disorder (BPD [2]). General psychiatric admission (GPA) is of uncertain therapeutic value amongst self-harming and suicidal individuals, and potentially harmful if lengthy and unstructured for those with BPD [3–5]. Specialized evidence-based services developed for these individuals at times they cannot keep themselves safe is often needed, but more often lacking. These services are particularly necessary in situations of unique vulnerability, such as suicidal crises. When suicide and severe self-harm are acute risks it is essential that services are offered in a compassionate manner that honours the human dignity of the person who is suffering [6, 7].

A report commissioned by Sweden’s National Self-harm Project examined the prevalence of self-harm among individuals receiving mental health services across 84 psychiatric settings from 15 cities [8]. The sample was comprised of participants aged 12 years and older. Results indicated that almost half of the participants currently receiving mental health services had self-harmed during the past 6 months, with one in six adults self-harming five or more times the last 6 months. Three out of four young women between the ages of 12 and 18 reported self-harming over the same time period. Of those who had engaged in self-harming behaviour, more than half had attempted suicide at least once during their lifetime [8]. For a sub-group of individuals, often with BPD, self-harming behaviours are frequent and risk for suicide is recurrent [9].

Over the last 20 years several psychotherapeutic interventions have evolved for the treatment of individuals with self-harm as well as BPD [3, 5, 10, 11]. However, during crises and associated increases in self-harm and suicidal ideation, recommendations for clinical care are still conflicting. For individuals with imminent suicidal ideation, without recurrent self-harm, the routine practice is to offer psychiatric admission to an inpatient unit [12]. For individuals with recurrent suicidal ideation and self-harm, often diagnosed with BPD, the risk for iatrogenic effects may be considerable. Lengthy hospital admissions without a clear treatment structure are associated with clinical and functional decompensation amongst this group [3–5]. Consequentially there is a clinical practice of avoiding inpatient admission of individuals with these presenting features. The absence of consensus amongst crisis management recommendations is a regular burden requiring strategic planning at junctures that would be better suited to the provision of seamless clinical care. Ideally, the situation would not require having to re-negotiate the entire process (to admit or not admit) on behalf of every acutely suicidal individual when they themselves feel out of control.

### What is BA?

The nature of the BA admission is goal-driven structured respite. A BA admission is not a stand-alone clinical intervention, nor is it unstructured rest. Rather, BA serves as a method of providing structured support to address the stress that is generating self-harming or suicidal escalation. The conditions, parameters and goals for adding BA to a treatment plan are negotiated with the individual and their care providers in advance of crises, effectively “striking while the iron is cold.” The aim in doing so is to support the individual’s autonomy in creating and maintaining control over their health care and their situation more generally, and to avoid power-struggles and decompensation when it is time to leave the BA. The negotiation process results in a contract describing how BA should be implemented for the individual based on their individual needs and preferences.

Over repeated experiences of BA, the structure and stabilizing aspects of BA generalizes to the individuals’ life outside of the hospital, leading to reduced reliance on acute and general psychiatric hospital admissions while remaining safe in times of crisis [13, 14]. By learning new ways of responding/not responding during crises, and staying safe regardless of current emotion, gains from psychotherapy engaged in concurrently with BA (treatment as usual: TAU) facilitates capability to manage subsequent crises without turning to self-harm and suicidal behaviour [15]. For individuals with BPD, BA can be offered concurrently with treatments such as Dialectical Behavior Therapy (DBT) or Mentalization-Based Therapy (MBT). In fact, adding BA to an existing treatment plan may enable those with BPD to remain in specialized evidence-based treatments delivered on an outpatient basis. Careful consideration, planning and support are part of developing the BA contract so that those participating are helped to continue to engage in important activities outside the hospital *during* BA. An example is continuing to attend DBT skills training groups during BA if the individual is already committed to that treatment on an outpatient basis.

### Brief Admission in the Netherlands

In the Netherlands, BA was initially developed [16] to manage the vulnerability of some individuals with complex mental illness towards frequent crises including escalation of suicidality. The purpose of BA was crisis management in general, and avoidance of lengthy inpatient admissions in particular. As a crisis management strategy, BA was rapidly adopted across most mental health service centers in the Netherlands. Helleman, Goossens, Kaasenbrood, and van Achterberg [13] conducted a phenomenological study involving 17 participants with lived experience of BA in the Netherlands. With respect to the key features of BA that were identified, participants endorsed: (i) the organization of the brief admission; (ii) the quality of the contact with a nurse over the course of BA; (iii) the restorative value of time out from daily life; and (iv) the experienced value of the intervention. Participants described appreciating how BA prevented the onset and escalation of self-harm and suicide attempts at times of high stress and distress. The nature of self-admission to BA was described as key to providing support at the exact time needed by participants. A key feature of BA is participant control over when the BA occurs, as well as its duration within the confines of short parameters, such as days [13]. Twenty years after its inception in the Netherlands, the Dutch Multidisciplinary Guidelines for Personality Disorders [17] recommended BA as a crisis management strategy for individuals with a BPD.

### Review of research on brief admission for crisis management of self-harming, suicidal, and borderline individuals

The majority of the literature on Brief Admission (BA) is concerned with short-term hospitalization of individuals in the acute stages of mental health crises related to substance and alcohol use disorders, psychosis or neurological events and the inpatient management of their sequelae. Aside from the Dutch literature summarized above, only two publications [18, 19] were identified as related to brief admission for self-harming, suicidal, and borderline individuals. These contributions to the literature and their similarities and differences to Brief Admission Skåne (BAS) are summarized as follows.

Siefert’s [18] Goal-Oriented Limited-Duration BPD Inpatient Treatment (GOLDBIT) is similar to BAS in that both are time-limited adjunctive initiatives rather than stand-alone therapeutic interventions/clinical therapies. Rather, GOLDBIT and BAS are intended to support longer-term outpatient clinical interventions, such as Dialectical Behavior Therapy (DBT) or Mentalization-Based Therapy (MBT). A planned short inpatient stay to regain structure and return to ongoing outpatient therapy is viewed by both Siefert [18] and our group as more desirable than losing membership in specialized longer-term outpatient therapy due to unplanned prolonged emergency psychiatric admission. Like BAS, in GOLDBIT an assessment of feasibility is conducted prior to determining whether it is an appropriate add-on to existing care. Also similar to BAS, GOLDBIT has “meta-goals,” such as articulating expectations, creating structure, and engagement with staff and life on the unit, with an aim of generalizing the of benefits of GOLDBIT to life outside of the admission [18]. Finally, both BAS and GOLDBIT have clear, pre-determined discharge dates, planned in advance to avoid power-struggles and decompensation at termination. A short admission in GOLDBIT is longer in duration than BAS (10 days compared to 3 days in BAS). A difference between GOLDBIT and BAS is that BAS is available for all self-harming and suicidal individuals, not those specifically diagnosed with borderline personality disorder (BPD) as in GOLDBIT.

The second publication relevant to BAS focused on the management of BPD [19], which is in alignment with the views and purpose of BAS except the broader inclusion of self-harming individuals into BAS both with and without BPD diagnoses. These authors noted that specialized, evidence-based treatments for BPD are preferable to lengthy or unstructured hospital stays or general treatment as usual. Where emergency admissions are involved to de-escalate suicidality, their recommendations were to keep admissions as short as possible, to reduce loss of stabilizing aspects of life such as employment, social contacts, and housing. Biskin and Paris also noted iatrogenic effects of lengthy and unstructured inpatient admissions as risk-factors for BPD individuals. There was no mention of the role of standardized brief admission, possibly because the place of publication was Canada, where brief admission is not a current feature of the mental health system. Nevertheless, theirs is an important paper both in acknowledging expert opinion that BPD be treated primarily on an outpatient basis^1^ (with the exception of specialized residential treatments, such as those pioneered by Bohus et al. [20–22]). As well, they generated recommendations for admissions that are consistent with BAS, specifically that admissions be: 1. Brief in nature; 2. Not intended to replace psychotherapy; 3. Used to manage suicidal crisis in the short term, and 4. Ideally a component of the best treatment for this population, which is specialized, evidence-based psychotherapy delivered on an outpatient basis with the exception of aforementioned specialized residential treatments. All of these features are consistent with BAS.

Brief Admission (BA) is a crisis management strategy that enables individuals to admit themselves to hospital without prior physician consultation at times when there is risk for increasing self-harm, escalating suicidality, and when efforts to stay safe are failing. The person seeking BA can decide for themselves when they need hospital admission to prevent decompensation of mental health functioning, for a short period (days) at a maximum frequency (admissions per month). The model has been used in the Netherlands for more than 30 years but has not yet been standardized or scientifically evaluated by randomized controlled trial. The Brief Admission Skåne (BAS) RCT upon which the current protocol is based contributes this data to the literature.

Participants in the BASRCT are randomized to receive either BA alongside their regular mental health services/treatment as usual (BAS + TAU) or treatment as usual (TAU). These comparators were selected to determine whether BA added to an existing treatment plan can reduce the number of days spent in general psychiatric admission and associated forced care incidents amongst a population who tend to be heavy consumers of mental health services. These comparators further allow the evaluation of whether, as in the Netherlands, access to BA alongside TAU is associated with more positive experience of mental health services, increased autonomy, and increased quality of life.

### BAS objectives

The primary aim of the BASRCT is to determine if BA can replace general psychiatric admissions (GPA) for individuals with complex mental illness with recent histories of self-harm, at acute risk for escalating self-harm or suicide. This will be determined by examining hospital data collected from clinical records pertaining to:

i. Number of days with BA admission
ii. Number of days with GPA to hospital
iii. Number of days admitted under forced (involuntary) psychiatric admission
iv. Frequency of involuntary care incidents (e.g. restraints, seclusion and involuntary medication)

The secondary aims of the BAS study are to determine whether BA can:

i. Increase the individual’s level of functioning in activities of daily life
ii. Increase the individual’s ability to cope effectively with life stress
iii. Reduce the individual’s global psychiatric symptoms
iv. Reduce the frequency of all self-harming behaviours including suicide attempts
v. Reduce the severity of self-harming behaviours;
vi. Serve as feasible risk and crisis management model in the care of self-harming individuals, who may also be at risk for suicide.

The measures used over the course of the RCT to address these research objectives and their associated outcomes are listed in Table 1.

**Table 1:** Summary of the BAS Measures

Data provider	Outcome variable	Questionnaires
Hospital records	N of days with general admission to hospital	-
	N of visits at the psychiatric emergency department	-
	N of days with involuntary admission to hospital as defined by LPT	-
	N of involuntary acts as defined by LPT (Act on compulsory psychiatric care)	-
	N of BAS	-
	N of days with BAS	-
Research persons	Self-harm	*Five Self-Harm Behaviour Groupings Measure* (5S–HM [34])
	Self-harm	*The Inventory of Statements About Self-Injury* (ISAS [35])
	Emotion regulation	*The Difficulties in Emotion Regulation Scale* (DERS [36])
	Coping strategies	*The Brief COPE* [37]
	Health and disability	*The World Health Organization Disability Assessment Schedule II* (WHODAS 2.0 [38])
	Developmental cognitive disabilities Alcohol consumption, and alcohol-related problems.	*Five questions* [39] *The Alcohol Use Disorder Identification Test* (AUDIT [40])
	Drug abuse and drug-related problems.	*The Drug Use Disorders Identification Test* (DUDIT [41])
	Client satisfaction with human services	*The Client Satisfaction Questionnaire* (CSQ [42])
	Client satisfaction with BAS	*Individual’s Experience Scale* (IES [23])
Clinicians	Clinician, administering BAS, satisfaction with BAS	*Clinician’s Experience Scale* (CES [23])
Psychiatrists	Psychiatric disorders	*The Mini-International Neuropsychiatric Interview* (*M.I.N.I. 6.0* [43])
	Personality disorders	*Structured Clinical Interview for DSM IV Axis II disorders* (SCID II [44])
	The severity of the patient’s illness	*Clinical Global Impression Severity* (CGI-S [45])

### Trial design

The BASRCT design is an interventional parallel randomized controlled trial with single-blinding. Participants are randomized at an individual level to either BAS + TAU or TAU. All participants are randomized one-to one (1:1).

## Methods: participants, interventions, and outcomes

### Study setting

*Psykiatri Skåne* is the provincial public mental health organization in the region of Skåne, South Sweden, providing individuals living in Skåne with psychiatric care. Region Skåne has approximately 1.3 million inhabitants and is served by four geographically organized adult psychiatric clinics. The geographically based organizations are served by one inpatient psychiatric hospital, and several outpatient clinics. Every inpatient setting has two to four wards treating individuals in the acute stages of primarily non-psychotic mental illnesses.

For the purpose of testing the effectiveness of Brief Admission Skåne (BAS) in the current RCT, a general psychiatric ward was chosen in each participating site by their management team comprised by clinical director, the psychiatrist with ultimate clinical responsibility at the unit and the heads of the local wards and units. To avoid drift of the effects of BA with treatment as usual (TAU), acute general psychiatric admission for participants included in the study was directed to units other than those providing BA whenever possible.

### Participants

***Inclusion criteria:***

- Current episodes of self-harm and/or recurrent suicidality
- Fulfilling at least three criteria for a diagnosis of BPD.
- Admitted to psychiatric hospital for acute care for at least seven days or presenting to the psychiatric emergency department at least three times during the last six months.
- Age 18–60 years.

***Exclusion criteria:***

- No regular contact with outpatient psychiatric services.
- Unstable housing
- Somatic disorder or need for medication management that significantly contributes to inclusion criteria (e.g. if self-harm only occurs during episodes of hypoglycemia in a diabetic person or in someone with substance-induced psychosis).

#### BAS randomized controlled trial: intervention

Brief Admission Skåne (BAS) refers to the current randomized controlled trial evaluating the effectiveness of a standardized version of brief admission (BA) for individuals with recurrent self-harm, escalating suicidality, and BPD. Within BAS, individuals can be admitted to hospital for a maximum duration of three consecutive days at a maximum frequency of three times per month. BAS is defined by these parameters as well as by the approach or demeanor of clinical staff, which should be warm, welcoming and bright.

Participants randomized to TAU will receive no intervention from the study protocol, except the baseline assessments and repeated assessments administered on the same schedule as described for the treatment group, every six months. They will not be given the evaluation measures that are specific to the BAS intervention (the IES and the CES [23]).

A broad spectrum of mental health services is comprised by TAU. These include psychopharmacological treatment and psychosocial interventions ranging from regular supportive contact with an out-patient clinician to time-specific specialized evidence-based treatments such as DBT and MBT. Involuntary and voluntary psychiatric admission (GPA) is used conservatively, in situations with very high risk for severe harm (for example, with a high probability of death) of self or others, or for specific treatment of co-morbid psychiatric disorders.

#### Criteria for discontinuing or modifying allocated interventions for a given participant

*Controlled by the participant:* The participant modifies the dose by choosing the duration and frequency of the BAs that they will add to their treatment plan, within the parameters of BAS (maximum three day admissions, up to three times a month.) If a participant tries BA and does not find it to be helpful, meaningful or if the participant finds BA harmful, they can choose to not initiate further BAs. If during a BA a participant wishes to terminate prior to the duration they planned in advance, the goal of the BA is reviewed, and the individual is asked to decide whether their desire to leave early is in the service of that goal. If a BA is terminated early, the outcome is evaluated afterwards with the individual and their care providers.

*Controlled by the clinician:* A BA is terminated following severe self-harm during BA, escalating suicidality during BA, or on occasions in which the participant has broken the terms of the contract signed prior to their first BA (for a sample contract please see [23]). If an adverse event involves clinical worsening with respect to self-harm or suicidality, the person is walked over to the Emergency Room and admitted as a general psychiatric inpatient. Care is taken to communicate the fact that a BA terminated early is a learning opportunity rather than a failure. The individual is warmly welcome back to the ward for another BA when they are ready to try again.

*Controlled by the clinician in collaboration with the PI and the research team:* If a participant engages in severe self-harm during BA, attempts suicide, or in situations in which other people at the ward were put at risk (adverse events), the BAS contract is put on hold until the individual can learn and express what skills they can use to avoid these outcomes in the future.

#### Strategies to improve adherence and fidelity

The National Institutes of Health Behavior Change Consortium (BCC) define treatment fidelity as methods and measures used over the course of implementation of an intervention to increase validity and reliability [24]. To monitor and ensure that BA “dose” content, and conditions were as adherent to the formulation of BA as possible, an objectively scored fidelity measure was developed [23]. The Brief Admission Skåne Fidelity Measure (BASFM) has accompanying rating forms to evaluate every fifth BAS negotiation^2^ that is videotaped for this purpose. Videotaped contract negotiations are then dubbed from Swedish into English for fidelity rating. M. Helleman and S. Liljedahl independently rate each videotaped negotiation, compare fidelity ratings, resolve any discrepancies in ratings between them, and give feedback to the principal investigator (S. Westling) who shares feedback directly with clinical staff delivering BAS. Where deviations to adherence are significant, emergency feedback is given at the earliest convenience of all parties. Inter-rater reliability of fidelity ratings will be calculated alongside the results of the BASRCT.

Further based upon the BCC treatment fidelity recommendations, parallel user experience measures were developed, for administration both to individuals receiving BA and to clinicians providing BA [23] within BAS. These measures are administered to individuals and their BA clinicians on every occasion that BA was delivered, as well as after every negotiation (every 6 months). Results of these measures were calculated at the conclusion of the pilot phase of BAS implementation. Both individual and clinician experience measures, as well as the procedure of data collection was streamlined to better reflect clinical reality on the wards as explained by clinical staff and individuals with lived experience receiving BA.

#### Outcomes

For a list of all primary and secondary outcomes including specific measurement variables, please see Table 2: All items from the World Health Organization Trial Registration Data Set.

**Table 2:** All items from the World Health Organization Trial Registration Data Set in relation to the Brief Admission Skåne Randomized Controlled Trial (BASRCT)

Data category	Information
Primary registry and trial identifying number	Clinicaltrials.gov NCT02985047
Date of registration in primary registry	November 25, 2016
Secondary identifying numbers	N/A
Sources(s) of monetary or material support	Mats Paulsson Stiftelse; Swedish Research Council; Sweden’s National Self-Harm Project (NSP); Regional Research Funds; Söderström-Königska Foundation; Ellen och Henrik Sjöbrings Minnesfond; OM Persson Stiftelse; Maggie Stephens Stiftelse
Primary Sponsor	Region Skåne Clinical Psychiatric Research Center Baravägen 1 22,185 Lund Sweden
Secondary Sponsor	None
Contact for public queries	Sophie Liljedahl Department of Psychology Lund University Box 213 221 85, Lund Sweden Phone: +46 0708 88 5618 Email: sophie.liljedahl@psy.lu.se
Contact for scientific queries	Sophie Liljedahl (contact information above) Sophie Westling, M. D., Ph. D. Lund University Box 213 221 85, Lund Sweden Phone:+46 0735 62 6099 Email: sophie.westling@med.lu.se
Public Title	Brief Admission Skåne (BAS) Brukarstyrd Inläggning (BI) – *Swedish*
Scientific Title	Brief Admission Skåne Randomised Controlled Trial (BASRCT)
Countries of recruitment	Sweden
Health condition or problem(s) studied	Individuals with three or more symptoms of borderline personality disorder; self-harming and/or suicidal individuals receiving public mental health services in the region of Skåne (South Sweden).
Intervention(s)	Brief Admission + Treatment As Usual OR Treatment As Ususal (BA + TAU OR TAU)
Key inclusion and exclusion criteria	Ages eligible: ≥ 18 years to 60 years. Sexes eligible: Individuals or any sex or gender Accepts healthy volunteers: no Inclusion criteria: adult patient (≥ 18 years to age 60), individuals receiving public mental health services in the region of Skåne, currently engages in self-harm and/or recurrent suicidal behavior, fulfills at least three criteria for a diagnosis of BPD, admitted to psychiatric hospital for acute care for at least 7 days or presenting to the psychiatric emergency department at least 3 times during the last 6 months. Exclusion criteria: No regular contact with outpatient psychiatric services, unstable housing, somatic disorder that significantly contributes to inclusion criteria
Study type	Interventional Allocation: randomized Masked: single blind Primary purpose: self-harm and suicide crisis and risk management
Date of first enrolment	October 2015
Target sample size	124
Recruitment status	Recruiting
Primary outcome(s)	1. Number of days with hospital admission Time frame: Change between the period from 12 months retrospectively to baseline and the period from baseline to 12 months prospectively. 2. Number of days with Brief Admission, general psychiatric admission, forced (involuntary) admission
Secondary outcome(s)	1. Frequency of forced acts (e.g. restraints and forced medication). Time frame: Change between the period from 12 month retrospectively to Baseline and the period from baseline to 12 months prospectively. 2. Individuals’ Experiences of the intervention. Time frame: Data collected at each Brief Admission during a period of 140 weeks. Outcome: Scores from the questionnaires developed for the method: *Individual’s Experience Scale (IES).* 3. Clinicians’ experiences of the intervention. Time frame: Data collected at each Brief Admission during a period of 140 weeks. Outcome: Scores from the questionnaires developed for the method: *Clinician’s Experience Scale (CES)* 4. Frequency of all self-harming behaviours including suicide attempts. Time frame: Change in frequency between baseline, 6 months and 12 months prospectively. Outcome: Scores from the *Five Self-Harm Behaviour Groupings Measure (5S–HM)* 5. Severity of self-harming behaviours Time frame: Change in severity between baseline, 6 months and 12 months prospectively. Outcome: Scores from the *Five Self-Harm Behaviour Groupings Measure (5S–HM)* 6. Level of functioning in activities of daily life. Time frame: Change in ratings between baseline, 6 months and 12 months prospectively. Outcome: Scores from the *World Health Organization Disability Assessment Schedule II (WHODAS 2.0)* 7. Ability to cope effectively with life stress. Time frame: Change in ratings between baseline, 6 months and 12 months prospectively. Outcome: Scores from *The Brief* COPE 8. Ability to regulate emotions Time frame: Change in ratings between baseline, 6 months and 12 months prospectively. Outcome: Scores on the *Difficulties in Emotion Regulation Scale (DERS)* 9. Global psychiatric symptoms. Time frame: Change in ratings between baseline, 6 months and 12 months prospectively. Outcome: Scores from the *Clinical Global Impression Severity Scale (CGISS)* 10. Satisfaction with health care Time frame: Change in ratings between baseline, 6 months and 12 months prospectively. Outcome: Scores on the *Client Satisfaction Questionnaire (CSQ)*

#### Participant timeline

**Fig. 1:**
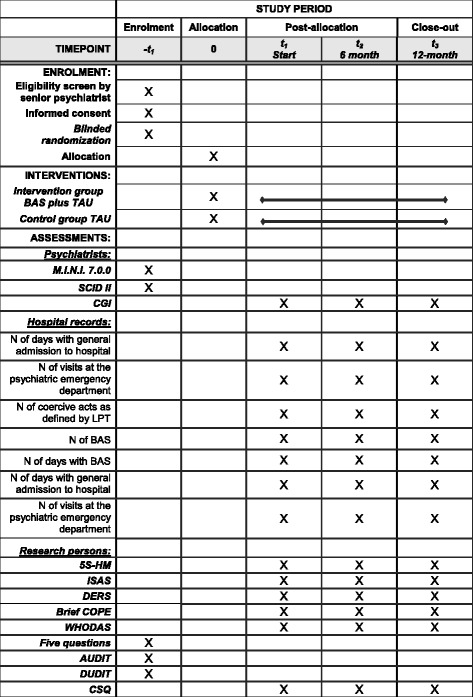
SPIRIT flow diagram: Schedule of enrolment, interventions and assessments

All participating staff at inpatient and outpatient units were given the inclusion and exclusion criteria for BAS and were instructed to ask prospective BAS candidates fulfilling these if they would like to participate in the BASRCT. If a prospective participant was interested, contact was made with the principal investigator (PI) of the study who requested consent to participate in BAS and conducted a psychiatric assessment to verify if inclusion criteria were fulfilled. A time schedule of enrolment, interventions, and assessments are presented in Fig. 1. A list of abbreviations is in Additional file 1: Appendix 1.

#### Sample size

Data collection is in progress, until the conclusion of the trial (June 2018). The online program G*Power, 3. 1. 7 [25] was used to calculate a priori power for analyzing main effects and interactions for an A X B mixed design where A is a between-subject factor with two levels (experimental and control groups) and B is a within-subjects factor with three levels (three repeated assessments). Assuming that three effects (i.e., between levels of the factor A, within levels of the factor B and within-between interaction A X B), are of medium size (f = 0.25; see [26]), a significance level is of α = .05, and the power values of the F tests are.85, a total of *N* = 98 must be recruited. Attrition in this population based on previous studies has been estimated to be approximately 25% [11, 27]. In order to attain the required sample size for these power estimate, including expected attrition, a total of *N* = 124 participants is required.

#### Recruitment

The first three months of the study (Oct, 2015–Jan, 2016) served as a pilot phase with the goal of optimizing the intervention, evaluating the inclusion and exclusion criteria and preliminary testing to determine whether the quality and quantity of assessments were adequate and feasible. The experience of receiving and delivering BA was evaluated with IES [23] and CES [23], respectively.

Data collection was suspended from January to March, 2016. During this phase every videotaped negotiation was translated and evaluated by the authors of the Brief Admission Skåne Fidelity Measure (BASFM [23]). Feedback from the evaluation measures (the IES and CES [23]) was extracted and reviewed by the senior researchers in this study to determine whether the content or procedures are functioning as anticipated, and to determine whether there are any areas in need of improvement. Substantial changes to any aspect of the study or its measures were sent to the Regional Ethics board (EPN Lund) for review. All requested changes (listed below) were formally approved:

- The contract was revised after feedback from participants and clinicians.
- Minor linguistic changes were made in the IES and CES after feedback from clinicians and participants.
- The procedure administering the IES and CES was revised in order to facilitate prompt evaluation.
- The BASFM [23] was revised over the course of the pilot phase. The most significant change was to evaluate only what could be objectively rated by video-tape, which was the negotiation process. Other changes occurred with phrasing and scoring of BASFM, to reduce cultural differences between interpretations of clinician warmth, and to give partial scores for items on the BASFM where some component of the item was present but other required components were missing.

Data collection for the active phase of the study began in March 2016 (baseline) and will terminate in June 2018.

## Methods: assignment of interventions

### Allocation

#### Randomization and sequence generation

Ethical approval for this study was received in 2014 (Dnr2014/570, Lund). In the current RCT (Brief Admission Skåne: BAS) participants are randomized at an individual level to either BA plus Treatment as Usual (TAU). Block randomization, using tables with random numbers with blocks of four are used in order to minimize the confounding effect of changes in general care over time. Randomization is stratified according to treatment site. Random number tables are generated using the web-based tool Research Randomizer [28]. The data will be handled with Intention To Treat (ITT) analysis, so that once participants are randomized, their data will be included in all analyses regardless of whether they drop out of the study prior to its termination. Data from all interview and self-report measures are collected in an encrypted online data management program housed at a local Swedish university.

For each participant in the BASRCT, hospital data are collected retrospectively from 12 months prior to baseline, over the course of the active phase of the study, and at six and 12 months post-baseline. Since access to BA requires a BA contract that is negotiated during a relatively stable phase involving the BA recipient as well as the outpatient clinician and a clinician from the BA unit, a delay can be expected between randomization and access to the intervention. In order to monitor the intervention group only when having access to BA, baseline for individuals randomized to the BA condition was set to the date that the BA contract was introduced and signed by the participant. For individuals randomized to the control condition, baseline was set to the date that screening assessment and randomization occurred.

#### Sequence allocation, implementation and blinding

The third author of this paper (D. D.) generated the allocation sequence, which was handled by a research nurse who prepared and sealed four series of consecutively numbered, sealed, opaque randomization envelopes. Numbers were generated one per site and strata, each envelope containing treatment instructions, as well as information on the procedures of data collection during the study. The PI (SW) enrolled the participants giving each of them a consecutive research number also generated one per site and strata. If the participant was eligible for the study SW assigned them by giving them a consecutive randomization envelope which was opened and signed by the participant. As the investigator was blind to the allocation, this study is a single blind trial.

## Methods: assignment of interventions

### Data collection methods

A complete description of assessment data and collection of outcome, baseline, and other trial data is summarized in Table 2: All items from the World Health Organization Trial Registration Data Set. The timeline of assessments is included in Fig. 1. SPIRIT flow diagram of enrolment, interventions and assessment. The BASFM, described above in the section on *Strategies to improve adherence and fidelity* has been published as component of the BASP [23].

### Processes to promote data quality: BAS training

A standardized 1-day workshop was developed to train clinical staff on the intervention and documentation encompassed by BAS, as well as a 1.5 h lecture for managers, physicians and administrators. This step was taken to follow fidelity recommendations from Bellg et al. [24] in relation to standardizing provider training, measuring provider skill acquisition post-training, and following up on how provider skills are retained over time. Accordingly, all staff at hospital wards where BA was going to be delivered received the 1-day workshop. Additional 60–90 min information on the BASRCT was offered to all staff working with self-harming, suicidal, and borderline individuals at the emergency wards, other inpatient wards, outpatient units, as well as a team of temporary relief staff. Outpatient clinicians working with the target group as well as relief staff were also encouraged to attend the 1-day workshop. The 1-day workshop was evaluated by attendees at the conclusion of each workshop. At every clinic a single information session for administrators and physicians was offered alongside two options to receive the 1-day workshops on how to deliver BAS to clinical staff. Newly employed staff and those involved in delivering BAS but having missed the BAS training were offered to attend workshops at other sites. After all sites had participated in the workshops and information sessions, new workshops and information sessions were offered every 6 months to ensure newly recruited staff were adequately trained in administering BAS. Information on how the BASRCT has progressed is shared with administrators and physicians in the form of yearly lectures. While BAS is ongoing, monthly consultation is offered to all staff working at wards where BA is being offered as part of the BASRCT. On-call supervision is also available for more urgent situations.

#### Data management

Interview data will be coded by trained research staff and entered into statistical packages for future analyses. Since data is not entered manually we will not employ the procedure of data double-entry. Encrypted data collection measures are generated from a data management system at Lund University created for this purpose (Sunet Survey).^3^ BASRCT data will be downloaded from Sunet Survey, saved on encrypted memory drives and stored in a locked cabinet in accordance with ethical guidelines for data management set by the regional ethical review board (EPN: Lund.)

### Statistical methods

All statistical analyses will be conducted on the full intent-to-treat sample and will be based on change in mean scale scores between baseline and 6- and 12-month post-assessments. For a complete list of primary and secondary outcomes, see Table 1.

Mixed-effect regression models with random slopes and intercepts will be used to test whether the change on outcome variables over time will be more pronounced for BAS + TAU group compared to the TAU-WL group (group × time interaction). Besides the interaction term, all models will also include the main effects of time (in weeks) and group. Socio-demographic variables found to differ between treatment and control groups at baseline or that predict change in outcome will be included in statistical analyses as covariates.

In order to the address potential effects of missing data, we will apply a pattern-mixture approach described by Hedeker and Gibbons [29] to test whether the addition of (1) the completer status, (2) the completer status × time interaction, and (3) the 3-way interaction between completer status, time and group would significantly contribute to the core model.

Effect sizes will be calculated both for those patients who completed the study and for the intention-to-treat population.

## Methods: monitoring

### Data monitoring

There are no competing interests associated with this study from either the authors or the sponsor. Because all data are monitored by the research group, a data monitoring committee is not needed. The PI reports all activities and significant deviations from the original protocol to the VKP (Region Skåne), the Swedish Research Council, Mats Paulssons stiftelse and the National Self-Harm project every six months, alongside national trainings presentations and international presentations describing BAS.

Interim analyses were conducted after the pilot phase of the study was complete. No further interim analyses were conducted and none are planned. The BASRCT will be terminated upon the full recruitment of *N* = 124 participants, based on a priori power analyses. Stopping guidelines are increasing self-harm, escalating suicidality and posing risk of harm to others on the ward amongst more than three individuals in the BAS + TAU condition.

### Harms

All adverse and unintended events are directly reported from the ward staff to the PI and recorded in a log for adverse and unintended events. Isolated acts of minor self-harm were handled within the BA parameters (conversation with the ward clinician at discharge and at the start of next admission about precipitating factors, analysis of the event, and discussion of how to prevent further acts of self-harm going forward). Severe self-harm or putting others on the ward at risk was handled by putting a hold on the BA contract. While the contract was on hold the individual was encouraged to learn new skills to prevent further adverse events during future BAs. As soon as the individual and the outpatient clinician had agreed that this was achieved, the contract was updated at which time the individual explained how the newly learned skills could help them avoid future self-harming behaviours, or avoid placing others in the ward at risk.

### Auditing

The PI and/or a trained research assistant visited all wards participating in the study on a monthly basis, speaking with ward staff regarding the procedures, offering consultation, giving feedback on adherence, as well as feedback regarding any contact with an individual receiving BA during the past month. This process was not independent from the BASRCT researchers, but it was independent from the VKP/Region Skåne.

### Protocol amendments

All aspects of the BASRCT protocol are published in Additional file 2: Appendix 3 of a manual developed from the BASRCT, including a theoretical background, training materials, measures developed for the BASRCT and the BASFM [23]. The protocol is updated alongside ethically approved changes to the BASRCT, which is further updated on a quarterly basis in the trial registry at ClinicalTrials.gov. The protocol version upon which this paper is based is Liljedahl, Helleman, Daukantaitė, and Westling [23].

### Consent and confidentiality

The procedures for obtaining consent from potential BASRCT participants are as follows:

1. Individuals with a clinical presentation suggesting that they may fulfill inclusion criteria are asked by their outpatient clinician, a clinician at the psychiatric emergency unit or a clinician working at one of the inpatient wards if they want to participate in the study. If the individual is interested in participating, the clinician shares contact information to the BASRCT PI or RA.
2. The PI or RA provides written and verbal information about the study, including time for questions to the prospective participant. The information is given either in person (if the prospective participant is on a ward) or on the telephone and by mail (if the prospective participant is at home).
3. If the prospective participant is still interested, the PI or RA schedules an appointment.
4. At this appointment, the PI offers to give verbal information again and actively asks if the prospective participant has new questions since the last contact regarding the BASRCT. After this the prospective participant is asked to sign the consent form (please see Additional file 2: Appendix 3), which is co-signed by the PI obtaining consent.

Part of the approved ethical application includes an *Information Letter* (Additional file 2: Appendix 3a) to prospective participants, describing all aspects of the BASRCT, including the intention to publish de-identified aggregate data both in press and at national and international scientific meetings and conferences. Consent forms are signed to indicate understanding of, and agreement with the right to confidentiality, safe storage of de-identified records that are confidentially shredded 10 years after study completion, details of participation in the trial, the ability to change ones’ mind about all aspects of study participation and adherence to data privacy laws. Informed consent authorized by a signed consent form (Additional file 2: Appendix 3b) is part of the process of becoming eligible to participate in the BASRCT.

In 2016 the PI of the BASRCT applied for and received an ethical amendment to our original approved application (Dnr2014/570, Lund) to recruit participants from the existing study to participate in a 5 year follow-up analysis of biomarkers. A separate consent procedure was developed for this ancillary study (described in the *Information Letter* in Additional file 2: Appendix 3c).

### Declaration of interests

The authors declare that they have no financial or other competing interests.

### Access to data

The authors of this paper are the members of the research team. They will all have access to a de-identified final trial dataset for publications completed by this research group. The datasets generated and/or analysed during the current study are not publicly available due to the fact that they contain personal health information, and are protected by laws that govern protection of personal health information. All data requests to the corresponding author would first be discussed amongst the research group and then vetted by the Chair of the Regional Ethics Board (EPN: Lund, Sweden).

### Ancillary and post-trial care

Participants are not paid for participating in the project, nor do they have arrangements for post-trial care or insurance since the intervention BA is being offered in addition to their general mental health services/treatment as usual, and is not associated with any risks.

### Dissemination policy

Key functions of dissemination and implementation research are increasing the likelihood that evidence-based interventions are taken up in practice and administered with fidelity [30]. The purpose of Brief Admission Skåne (BAS) is to contribute a first step in establishing effectiveness of BA + TAU over TAU through the current BASRCT. We are mindful that subsequent research evaluating this method is required prior to its designation as an evidence-based practice [31–33]. Nevertheless, we wanted to follow best practices for enhancing treatment fidelity as a step towards feasible implementation and dissemination, both within the trial and going forward after its termination.

We are an international research group, facing the aforementioned problems of clinical management of crises related to escalating self-harm and suicide in individuals with and without borderline personality disorder. Clinical management of these crises is problematic in every one of our home countries with the exception of the Netherlands, where crises are curtailed by BA, which is a feature of the Dutch mental health system. Accordingly, we were mindful of the vitality of implementation and dissemination especially for international export from the early planning stages of BAS.

Our in-person observation of BA in the Netherlands generated the hypothesis that the clinical approach or “demeanour” of staff providing BA is key to its popularity amongst people receiving mental health services as well as its broad implementation in the Netherlands. Given that “clinical demeanour” is a challenging construct to operationalize, and that it can be easily interpreted subjectively, our focus was on measuring fidelity from before the BASRCT began. For this reason, the best practices and recommendations from the National Institutes of Health and Behaviour Change Consortium (BCC [24]) guidance were followed as closely as possible for enhancing treatment fidelity in the BASRCT. If our primary hypotheses are supported, that is, if BAS can replace general psychiatric admissions for individuals with self-harm at acute risk for suicide, our efforts in implementation and dissemination will continue with an aim for BAS to be merited as a recognized evidence-based intervention. To facilitate international export, English language Train-the-Trainer manuals are being developed, and the BAS fidelity measure and the BAS protocol including clinician training materials have published towards this purpose [23].

### Roles and responsibilities and authorship eligibility

1. SW is the PI of the study and has been involved in all aspects of study development and implementation. She is the principal and in some cases sole recipient of funding for the RCT and she is primarily responsible for data acquisition, training and supervision of clinicians and administrators participating in the RCT. She contributed to the BASRCT protocol.
2. SL contributed to the conception of the study, was involved in drafting the manuscript and revising it critically for important intellectual content; she principally developed the fidelity measure and BASRCT manual, protocol, and fidelity measure, and will train others in coding BAS fidelity. She contributed to all aspects of study development and principally developed the manuscript and its revisions.
3. MH contributed to the conception of the study and co-developed the fidelity measure and manual. She contributes to training and supervision of clinicians and administrators participating in the RCT and has contributed original content to the manuscript and its revisions. She contributed to the BASRCT protocol.
4. DD made essential contributions to conception and study design and is responsible for data analysis and interpretation of data and writing results. She contributed original content to the manuscript and its revisions. She contributed to the BASRCT protocol.
5. ÅW agreed to be accountable for all aspects of the work in ensuring that questions related to the accuracy or integrity of any part of the work are appropriately investigated and resolved.

All authors read and approved the final manuscript. No professional writers were used.

## Discussion

Brief Admission (BA) is an adjunctive treatment in which individuals can engage alongside specialized evidence-based psychotherapy offered on an outpatient basis. As a concurrent structured respite plan, BA has been well received by individuals and care providers for more than 20 years in the Netherlands [17] as a crisis management approach for individuals with a BPD. The Brief Admission Skåne (BAS) randomized controlled trial upon which the current protocol is based represents the first international effort to standardize, export, and implement BA into a different language, cultural setting, and country from which it was developed.

Implementing BA in a general psychiatric setting is an extensive and time-consuming process. All staff and management at the participating psychiatric wards, emergency departments and outpatient units must be informed of the availability of BA, be trained in the referral process, and understand BA parameters and implementation well enough to either explain it adequately to prospective participants or deliver BA themselves. Importantly, all professionals must also be willing to initiate and maintain good collaboration with the BA ward, the BAS PI and RA, and amongst each other.

Implementing BA to a novel setting outside of its country of origin has required regular information-sharing, education and consultation to each entire setting in which BAS has been implemented by a full-time PI and a part-time RA. Daily communication between the PI and RA and regular communication with the wards involved in BAS implementation was required in order to maintain adherent BA delivery, as well as to promptly solve problems as they arose. Regular (monthly, and if needed weekly) consultation as well as repeated (at least 2 times a year) training on BAS has been offered over the duration of the trial. Practical and clinical emergencies related to suicidality or other crises arising during BA were managed by direct clinical supervision with the PI, who is a psychiatrist, on an on-call basis. In more complex clinical situations, urgent consultation amongst the research team, the majority of who are also clinicians, was required so that adherent decisions could be made by consensus. Although BA itself is relatively simple compared to specialized evidence-based treatments for this population, the coordination and collaboration surrounding its adherent implementation is resource and time-intensive. An essential facilitating factor has been ongoing collegial and healthy collaborations within the BASRCT, the vitality of which cannot be overstated.

At the time of writing this paper no meta-analyses, randomized controlled trials or controlled trials were published testing the efficacy or effectiveness of BA as a replacement for GPA amongst self-harming and suicidal individuals with three or more criteria of borderline personality disorder. In this manner, the BASRCT upon which the current protocol is based represents a unique contribution to the literature. The current BASRCT is the first step in the direction of generating data to determine its effectiveness.

## Additional files

Additional file 1: Appendix 1 - List of abbreviations. (DOCX 19 kb)

Additional file 2: Appendix 3 - Model consent forms and other related documentation given to participants and authorized surrogates. Plans for collection, laboratory evaluation, and storage of biological specimens for genetic or molecular analysis in the current trial and for future use in ancillary studies. (DOCX 29 kb)

## Footnotes

1 This was first attributed to Linehan [3]

2 The negotiation process is a conversation that precedes entry into BA at which time every aspect of BA is explained to prospective candidate, preferences are recorded, questions are answered, and BA contracts are signed. The signed contract becomes part of the person’s care plan and hospital record.

3 For more information about Sunet Survey please see: http://www.staff.lu.se/research-and-education/education-support/quality-enhancement/sunet-survey-a-survey-tool
