# Supplementary material for: A standardized crisis management model for self-harming and suicidal individuals with three or more diagnostic criteria of borderline personality disorder: The Brief Admission Skåne randomized controlled trial protocol (BASRCT)
Source: BMC Psychiatry. 2017 Jun 15;17:220. doi: 10.1186/s12888-017-1371-6 (PMC5472925; doi:10.1186/s12888-017-1371-6)
Supplement: Supplementary file 1 — Appendix 1 - List of abbreviations. (DOCX 19 kb) [file 12888_2017_1371_MOESM1_ESM.docx]

**APPENDIX 1: List of abbreviations**

5S-HM =Five Self-Harm Behaviour Groupings Measure

AUDIT = The Alcohol Use Disorder Identification Test

BA: Brief Admission
BASFM: Brief Admission Skåne Fidelity Measure
BAS: Brief Admission Skåne
BCC: National Institutes of Health Behaviour Change Consortium
BPD: Borderline personality disorder
CES: Clinician Experience Scale (BAS measure)

CGI-S = Clinical Global Impression Severity

CSQ = The Client Satisfaction Questionnaire
DBT: Dialectical Behavior Therapy

DERS = The Difficulties in Emotion Regulation Scale
Dnr: Diary Number; unique identifier of a registered ethical application that has been reviewed by the Lund Regional Ethics Review Board^[[1]](#footnote-1)^
EPN: Regional ethics review board (Lund)

DUDIT = The Drug Use Disorders Identification Test

GPA: General psychiatric admission
GOLDBIT: Goal-oriented limited-duration BPD inpatient treatment
IES: Individual Experience Scale (BAS measure)
ISAS = The Inventory of Statements About Self-Injury

ITT: Intention-to-treat
LPT: Lagen om Psykiatrisk Tvångsvård. Swedish Mandatory Mental Care Act^[[2]](#footnote-2)^.

MBT: Mentalization-Based Therapy

M.I.N.I. 7.0.0 = The Mini-International Neuropsychiatric Interview
PI: Primary investigator
RA: Research Assistant

RCT: Randomized controlled trial

SCID II = Structured Clinical Interview for DSM IV Axis II disorders
TAU: Treatment as usual

VKP: Vetenskapcentrum för klinisk psykiatri, Lund
WHODAS 2.0 = The World Health Organization Disability Assessment Schedule II
WL: Waiting list

1. <http://www.epn.se/lund/om-naemnden/> [↑](#footnote-ref-1)
2. The law in Swedish is retrieved at:

   <http://www.riksdagen.se/sv/dokument-lagar/dokument/svensk-forfattningssamling/lag-19911128-om-psykiatrisk-tvangsvard_sfs-1991-1128>

   Information in English can be retrieved at <http://www.domstol.se/Funktioner/English/Matters/Compulsory-care/Compulsory-mental-care/> [↑](#footnote-ref-2)
