# Supplementary material for: A standardized crisis management model for self-harming and suicidal individuals with three or more diagnostic criteria of borderline personality disorder: The Brief Admission Skåne randomized controlled trial protocol (BASRCT)
Source: BMC Psychiatry. 2017 Jun 15;17:220. doi: 10.1186/s12888-017-1371-6 (PMC5472925; doi:10.1186/s12888-017-1371-6)
Supplement: Supplementary file 2 — Appendix 3 - Model consent forms and other related documentation given to participants and authorized surrogates. Plans for collection, laboratory evaluation, and storage of biological specimens for genetic or molecular analysis in the current trial and for future use in ancillary studies. (DOCX 29 kb) [file 12888_2017_1371_MOESM2_ESM.docx]

**APPENDIX 3a
*Information letter preceding signed consent to participate in the BASRCT***

**Information for Potential Research Participants**

**Brief Admission Skåne: Comparison between Brief Admission alongside Treatment as Usual versus Treatment as Usual for people with self-harm at risk of suicide**

**Request to participate:**You are asked if you want to participate in the research study because you have sought services in Psychiatry or have been admitted to a psychiatric ward in the past year due to mental illness, harming yourself or attempting suicide.

**Background and purpose:**There is insufficient knowledge about what psychiatric treatment produces good results if you suffer from mental illness and have severely harmed yourself. This includes why and when you should be admitted to a psychiatric ward. We know that general psychiatric admission rarely helps you feel better and therefore should be avoided. However, we also understand that these same admissions may be necessary to avoid serious injury or save the lives of those who have suicidal behaviours. Since health care providers often want to avoid hospitalization, conflicts can easily arise between the person seeking help and the health care provider in the emergency department. We want this research study to examine whether Brief Admission can replace regular admission/treatment as usual for people at risk of harming themselves or attempting suicide. Brief Admission means that you are able to admit yourself to the hospital for a short time, without your reason for wanting hospitalization evaluated by a doctor. The method has been well used in the Netherlands for 30 years. It has not yet been tested on a large scale in Sweden or elsewhere.

**How does the research study work?**If you give your consent to participate, you meet a senior psychiatrist and complete a thorough diagnostic interview, which is estimated to take two to three hours. If the psychiatrist thinks you need further assessment after this you will be given a referral. The type of research study being conducted is called a randomized controlled trial (RCT), which provides the most reliable results when testing whether a treatment is effective. It is randomized, meaning that you will be been randomized to either access Brief Admission in addition to your regular treatment, or that you are been randomized to just get your regular treatment (Treatment as Usual).

**If you are randomized not to receive Brief Admission**You will be interviewed by a research assistant at the beginning of the research study and fill out forms about functioning in everyday life and managing crises and emotions. You will also be asked to complete questions regarding whether, how, and how often you have harmed yourself. The research assisstant will aid in the evaluation of the severity of your mental illness. After your first meeting, you will meet the research assistant 6 and 12 months months later. The research assistant will then estimate whether there have been any changes in your symptoms. Each examination is expected to take 60-90 minutes and will take place in the clinic where you normally receive outpatient care. In addition, data will be recorded regarding how many times a day you have been admitted or sought emergency care, and if you have received involuntary or coercive measures during the study and for a period of twelve months prior to your participation. Your participation in the study will last for a total of 12 months.

**If you are randomized to be offered Brief Admission**You will be interviewed by a research assistant at the beginning of the research study and fill out forms about functioning in everyday life and managing crises and emotions. You will also be asked to complete questions regarding whether, how, and how often you have harmed yourself. The research assistant will aid in the evaluation of the severity of your illness and you will be asked to complete an evaluation of how Brief Admission worked for you in the past month. This will be repeated after 3, 6 and 12 months, when a research assistant also will also evaluate whether there have been changes in the big picture of your mental health. Time estimated to complete the evaluations is 60-90 minutes, in the clinic where you receive outpatient care. In addition, data will be recorded how many times a day you have been admitted or sought emergency care, and if you have received involuntary or coercive measures during the study and for a period of twelve months prior to when your participation begins.

After you have met the research assistant for the first time, treatment is started with Brief Admission. You will work with your primary mental health and the research assistant to go through the attached contract where it is described how the Brief Admission process works, as well as the conditions required for admission. You will have possible to discuss the contract with the research assistant. During this initial conversation, you will also discuss the function Brief Admission can have in your of treatment. The aim is that Brief Admission should be able to be a part of your treatment plan. This initial conversation will take an hour and, if necessary, additional visits booked in for follow-up discussion. At every opportunity you use Brief Admission, the experience will be evaluated by both you and a research assistant to evaluate how it worked, and if necessary, determine how to improve your experience in the future. In addition to Brief Admission, you will be offered the very same treatment as before. You are entitled to the same medication, the same therapist and the same opportunity to seek emergency admission as if you choose not to participate in the study.

Your participation in the study will last for a total of 12 months.

**What are the risks:**Because Brief Admission does not replace any part of the care you are offered today, you will have access to the same care now no matter what group you are randomized to. Therefore, there is no increased risk associated with the research study, other than that you may get tired of answering questions and surveys.

**What are the advantages:**Whether you are randomized to Brief Admission or not, you will get a review of the psychiatric evaluations you have previously completed. These will be supplemented so that you will have received a thorough psychiatric assessment at the outset of the research study. If you are randomized to Brief Admission, you will have more opportunities to be hospitalized, without your reasons for hospitalization being evaluated by a doctor. This is something offered to you in addition to the care that you normally have access to, and does not replace any part of the care you are offered today. Past experience with Brief Admission has found that individuals with access Brief Admission received less coercive care and had a more positive experience of their mental health services than before. This is what we now want to investigate further.

**Data and privacy:**Your name and your contact information will be stored separately from the questionnaires and other information about you we collect over the course of the study. All information generated for the study is identified by a code that can be linked to your identity via a code key stored in a locked, fireproof safe, separate from your answers. This is to ensure your privacy, so that no information, directly or indirectly, can be traced to you. Only principal researchers will have access to your name and your contact information together with Psychiatry Skåne, as they are responsible for the safety of the study. The personal data in the form of hospital stays and forced care acts will be taken from your medical records and other information from the questionnaires you answer. The information will be stored for 10 years after the research study is completed and then destroyed confidentially. Your answers and your results of the questionnaires you provide will be treated according to the Personal Data Act (1998: 204), to prevent unauthorized access to them. According to law § 26 of *The Personal Data Act*, you have the right to request a copy of your personal health information, as well as information regarding how we manage your personal health information. You also have the right, under the law 28 § *per-Data Act* to request a correction of the personal data we hold about you. The Board of Psychiatry Skåne is responsible for your personal data. It is possible to contact the principal researcher (please see the contact information below) with a written request for your records. If you want to turn to a third party with questions or complaints, do not hesitate to turn to the Data Protection Officer, Region Skåne, 291 89 Kristianstad.

Only de-identified data will be used in the analyses in this research study. All results are reported on a grouped basis. The results will be reported in scientific international journals and conferences in order to disseminate knowledge as widely as possible.

**How do I get information on research results of the study?**You are welcome to contact Sofie Westling (contact information below), the lead researcher, if you want access to your personal information as well as results of the study.

**Insurance, compensation**Patient Injury insurance covers this. We do not pay participants for participating in the project.

**Voluntary**Participation in the research study is voluntary and does not affect the rest of your treatment. You have the right at any time during the research study cancel your participation without explaining the reason. If you choose to end your participation in the study this decision will not this affect the rest of the care that you receive. If you want to terminate your participation, please contact Sofie Westling (contact details below).

**Principal Investigator**If you have questions about the research or request access to your personal information, please refer directly to the principal investigator (contact details below). If you choose to initiate contact by email, please provide a telephone number as the confidentiality rules of the Region of Skåne prevent reply via e-mail.

Sofie Westling, MD, Ph.D. Psychiatrist
DBT team, Rehab Unit
Baravägen 1
22185 Lund
tel: 046-174953
e-mail: [sofie.westling@skane.se](mailto:sofie.westling@skane.se)

**APPENDIX 3b
*Consent form signed to participate in the BASRCT***

**Consent Form for participation in**

**Brief Admission Skåne: Comparison between Brief Admission alongside Treatment as Usual versus Treatment as Usual for people with self-harm at risk of suicide**

I have received information about the study Brief Admission Skåne: Comparison between Brief Admission and Treatment as Usual versus Treatment as Usual for people with self-harm and risk of suicide.

I have had the opportunity to ask questions and I have received answers to any questions that I might have had.

I give my consent to participate in the study, which means that my personal information is collected and processed by the researchers responsible for the study.

I also understand that I have the right at any time to withdraw my participation in the study. I do not need to give a reason if I choose to withdraw.

Date_________________

Name___________________________________________________________

Signature_________________________________________________

Study ID number___________________

Person who obtained consent_________________________________________________

Signature_________________________________________________

**APPENDIX 3c
*Information letter preceding signed consent to participate in the BASRCT***

**Information for Potential Research Participants**

**Brief Admission Skåne: Comparison between Brief Admission alongside Treatment as Usual versus Treatment as Usual for people with self-harm at risk of suicide
*Long-term follow-up of individuals with self-harm***

## Request to participate:

As a participant in the Brief Admission Skåne Randomized Controlled Trial (BASRCT), you are invited to participate in a long-term follow-up study relating to the biology of self-harm.

## Background and purpose:

Both people who suffer from repeated self-harm and their close relatives may be struggling with the question of how their situation will be in the future, as well as when and how their mental health will improve. Today there are no studies investigating how people with repeated self-harm fare in the long-term. Further, there are currently no psychopharmacological interventions that have reliably good results on managing self-harm. In order for these to develop, increased knowledge about the biological background to self-harm is needed. In order to develop knowledge regarding how people who self-harm feel over time, all participants in the BASRCT will be asked if they want to participate in a follow-up study that involves annual follow-ups for five years. In order to increase knowledge about the biological background of self-harm, study participation will involve taking blood samples to analyze biological factors that may be associated with self-harm.

How does the research study work?
If you give your consent to participate in this ancillary biomarkers study, you will be called annually for a visit where you fill out forms, similar to those you completed during the BASRCT. You will also receive a referral for leaving blood samples. Blood samples will be stored in the biobank in Region Skåne. From the blood samples we will then analyze biological and genetic markers related to inflammation, stress and how the cells of the body function. Samples will also be saved for possible future gene analyses, but these analyses will be specified and approved in a new application to the Ethics Review Board that would require your renewed consent. Your participation in this ancillary study lasts for a total of five years. After five years, a senior psychiatrist will conduct a new diagnostic review, similar to when you joined the BASRCT.
**What are the risks:**

There are no increased risks associated with participation in this ancillary biomarkers research study apart from being tired of answering the questions and experiencing minor physical pain due to blood sampling.
**Are there any advantages:**

There are no direct benefits to participating in the study, other than a possible sense of meaning or purpose from participating in a project may lead to increased knowledge on self-harm and its more effective treatment.

Data and privacy:
Your name and your contact information will be stored separately from the questionnaires and other information about you we collect over the course of the study. All information generated for the study is identified by a code that can be linked to your identity via a code key stored in a locked, fireproof safe, separate from your responses. This is to ensure your privacy, so that no information, directly or indirectly, can be traced to you. Only principal researchers will have access to your name and your contact information together with Psychiatry Skåne, as they are responsible for the safety of the study.

Personal data in the form of hospital stays and forced care acts will be taken from your medical records alongside other information from the questionnaires you answer. This information will be stored for 10 years after the research study is completed and then destroyed confidentially. Your answers and your results of the questionnaires you provide will be treated according to the *Personal Data Act* (1998: 204), to prevent unauthorized access to them.

According to law § 26 of *The Personal Data Act*, you have the right to request a copy of your personal health information, as well as information regarding how we manage your personal health information. You also have the right, under the law 28 § *per-Data Act* to request a correction of the personal data we hold about you. The Board of Psychiatry Skåne is responsible for your personal data. It is possible to contact the principal researcher (please see the contact information below) with a written request for your records. If you want to turn to a third party with questions or complaints, do not hesitate to turn to the Data Protection Officer, Region Skåne, 291 89 Kristianstad.

Only anonymous data will be used in the analyses. All results are reported on a grouped basis. The results will be reported in scientific international journals and conferences in order to disseminate knowledge as widely as possible.

How do I get information on research results of the study?
You are welcome to contact Sofie Westling (contact information below), the lead researcher, if you want access to your personal information as well as results of the study.

Insurance, compensation
Patient Injury insurance covers this. We do not pay participants for participating in the project.

Voluntary
Participation in the research study is voluntary and does not affect the rest of your treatment. You have the right at any time during the research study cancel your participation without explaining the reason. If you choose to cancel the research study will not this affect the rest of the care you receive. If you want to cancel your participation, please contact Sofie Westling (contact details below).

Principal Investigator
If you have questions about the research or request access to your personal information, please refer directly to the principal investigator (contact details below). If you choose to make contact by email, please provide a telephone number as the Regions of Skåne confidentiality rules prevent reply via e-mail.

Sofie Westling, MD, Ph.D. Psychiatrist
DBT team, Rehab Unit
Baravägen 1
22185 Lund
tel: 046-174953
e-mail: [sofie.westling@skane.se](mailto:sofie.westling@skane.se)
